# Fingerprint Identification Using SIFT-Based Minutia Descriptors and Improved All Descriptor-Pair Matching

**DOI:** 10.3390/s130303142

**Published:** 2013-03-06

**Authors:** Ru Zhou, Dexing Zhong, Jiuqiang Han

**Affiliations:** 1 Institute of Technology, Department of Communications and Integrated Systems, Tokyo 152-8550, Japan; E-Mails: zhouru19850319@gmail.com (R.Z.); kunieda@vlsi.ss.titech.ac.jp (H.K.); 2 School of Electronic and Information Engineering, Xi'an Jiaotong University, Xi'an 710049, China; E-Mail: jqhan@xjtu.edu.cn

**Keywords:** fingerprints, narrow sensor, SIFT, All Descriptor-Pair Matching, FISiA

## Abstract

The performance of conventional minutiae-based fingerprint authentication algorithms degrades significantly when dealing with low quality fingerprints with lots of cuts or scratches. A similar degradation of the minutiae-based algorithms is observed when small overlapping areas appear because of the quite narrow width of the sensors. Based on the detection of minutiae, Scale Invariant Feature Transformation (SIFT) descriptors are employed to fulfill verification tasks in the above difficult scenarios. However, the original SIFT algorithm is not suitable for fingerprint because of: (1) the similar patterns of parallel ridges; and (2) high computational resource consumption. To enhance the efficiency and effectiveness of the algorithm for fingerprint verification, we propose a SIFT-based Minutia Descriptor (SMD) to improve the SIFT algorithm through image processing, descriptor extraction and matcher. A two-step fast matcher, named improved All Descriptor-Pair Matching (iADM), is also proposed to implement the 1:N verifications in real-time. Fingerprint Identification using SMD and iADM (FISiA) achieved a significant improvement with respect to accuracy in representative databases compared with the conventional minutiae-based method. The speed of FISiA also can meet real-time requirements.

## 1. Introduction

**Figure 1.**
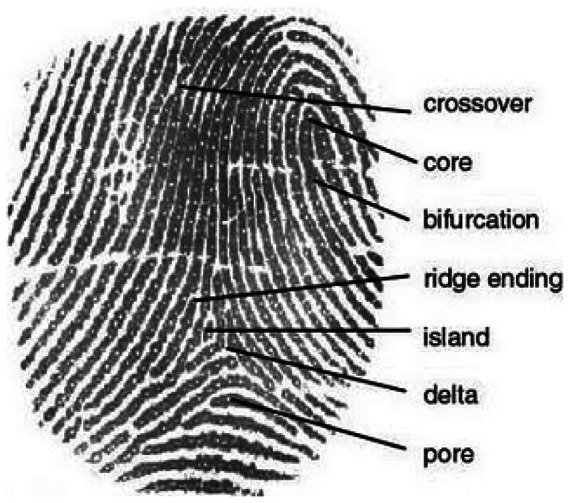
Fingerprint Structure.

Fingerprints have been used as a personal identification tool for a long time because of their uniqueness and time invariance. A fingerprint comprises ridges and valleys that correspond to the dark and white areas in the gray-level image. Figure 1 shows the structure of a fingerprint which includes examples of a crossover, core, bifurcation, ridge ending, island, delta and pore.

**Figure 2.**
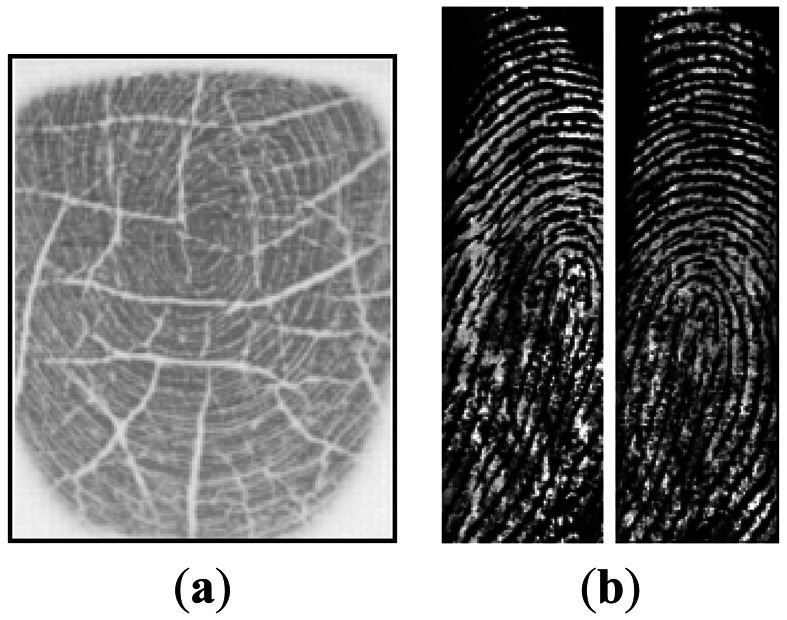
Example of fingerprint (**a**) with lots of cuts; (**b**) captured by a narrow-swiped sensor.

The most widely used fingerprint matching method is the minutiae-based matcher. The matcher performs fairly accurate fingerprint matching for minutiae-based verification systems [1–3]. However, the system has a number of disadvantages. Firstly, a minutia shape, which is a ridge shape associated with a minutia, can be cut off by cuts or scratches. The small cuts or scratches can be recovered by the methods used in previous research such as a Gabor-filter. However, it is very difficult to recover the ridges of a dry fingerprint which has lots of cuts. This can increase the ambiguity of minutiae when ridge shapes are used. One example of a fingerprint with lots of cuts is shown in Figure 2(a). A similar discussion of this challenge can be found in [4]. Secondly, the performance of this system will degrade significantly if the overlapping area between the template and the input fingerprint image is small, and when the number of available minutiae is few. This case occurs when a large translation of finger position occurs or when a swipe sensor with a very narrow width is used. One example is shown in Figure 2(b). Both images were captured by a swipe sensor with a width of 126 pixels. The actual number of overlapped minutiae between the template and input fingerprints is only approximately 10 for this kind of sensor. This paper proposes a new fingerprint verification algorithm using SIFT-based minutiae descriptor (SMD). The proposed method can successfully solve the two challenges mentioned above.

To reduce the ambiguity of minutiae, additional information can be attached to a minutia to form a descriptor. Several previous studies have utilized descriptors to improve accuracy. These mainly include ridge-based [3,5], orientation-based [6–8], minutiae-based approaches [9]. Ridge-based descriptors use one associated ridge of minutia as the additional information. An orientation-based descriptor is composed of the local ridge orientation at a set of sampling points around minutia. Minutiae-based descriptors use the information of neighboring minutiae as secondary features. Tico [8] sorted neighboring minutiae with respect to distance or angle in the local polar coordinate system, and the feature vectors consist of relationships between the first m neighboring minutiae and the central minutia.

The minutia descriptor proposed in this paper employs SIFT [10,11] information as the additional data in order to reduce the complexity of feature correspondence. The original SIFT algorithm proposed by Lowe [10,11] is an approach for extracting distinctive invariant features from images. The original algorithm has been successfully applied to a variety of computer vision problems based on feature matching, which includes object recognition, pose estimation, and image retrieval, *etc.* In recent years, the SIFT feature points have already shown their efficacy in other fields of biometrics including palmprint verification [12] and face verification [13,14]. Thus, the method is met with high expectations in fingerprint verification. The study of Park [15] showed the experimental results of applying SIFT [10,11] to fingerprint verification. However, his work has some disadvantages which lead to poor performance in terms of both accuracy and speed. Firstly, fingerprint images were insufficiently preprocessed in the image. The histogram equalization which was applied in [15] was inadequate because it can lead to different brightness in different regions of fingerprint images. Secondly, the original SIFT matcher is unsuitable for fingerprint verification because a fingerprint primarily comprises parallel ridges or valleys, thereby making the features less distinctive. Thirdly, the low computational efficiency is a hindrance to practical applications.

The proposed technique uses key points of SIFT for fingerprint verification. The proposed algorithm has several advantages over previous SIFT-based methods. Firstly, the proposed approach utilizes proper image processing to make the SIFT feature extraction robust against variations attributable to different finger pressures and noises. Secondly, the SIFT matcher is optimized for fingerprint verification based on a Hough Transform to expand the fingerprint images into large rotation cases. Thirdly, in order to enable the recognition system to perform in real time, a two-step fast matcher is proposed.

The rest of this paper is organized as follows: Section 2 presents definition of SIFT-based minutia descriptor (SMD). Section 3 describes the procedures for the two-step fast matcher, called improved All Descriptor-Pair Matching (iADM). Section 4 presents the experimental results and analysis to confirm the validity of the proposed method, named Fingerprint Identification using SMD and iADM (FISiA). Section 5 gives a brief conclusion.

## 2. SIFT-Based Minutia Descriptor (SMD)

In this section, we present the flow of image processing, minutiae extraction and the definition of SIFT-based Minutia Descriptor.

### 2.1. Fingerprint Image Preprocessing

**Figure 3.**
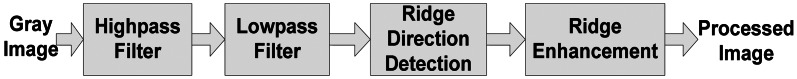
Image processing flow.

The SIFT descriptor becomes unstable in the presence of variations in finger pressure or differences in skin characteristics. Therefore, the gray-scale fingerprint images without pre-processing are not proper for original SIFT extraction. Filters are used to process the original fingerprint image to derive an enhanced gray image. Figure 3 shows the image processing flow employed in FISiA. It can be partitioned into the following major stages: highpass filter, lowpass filter, ridge direction detection, and ridge enhancement.

**Figure 4.**
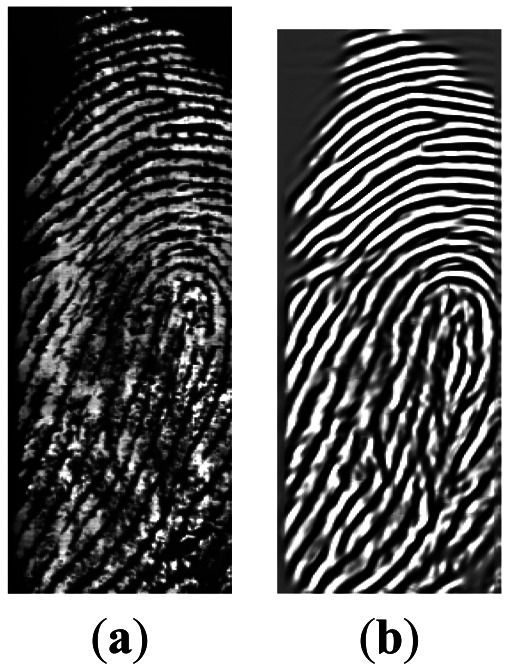
Effect of image processing on SIFT: (**a**) Original; (**b**) After enhancement.

The highpass filter is used to perform the brightness calibration. If the gray value of the image at position *(x,y)* is denoted by *I(x,y)*, the calculation of highpass filter *I_H_(x,y)* is computed in Equation (1) where the size of highpass window *k* is selected as 16 and the bias value *b* equals 128. It calculates average intensity within *k* × *k* window and subtracts average from the center pixel biased at *b*. The lowpass filter *I_L_(x,y)* is used to decrease noise as described by Equation (2), where the sizes of two lowpass windows *m* and *n* are selected as 4 and 2, respectively. The reason that lowpass filter uses two windows and uses the average of two averages is that the pixel in the smaller window is more similar to the target pixel. After accurate ridge detection for each pixel using look-up table is performed, the ridge direction detection for each block (8 × 8) can be estimated. The details can be found in [16]. The ridge enhancement [17] with a Gabor-like filter can be performed to enforce the fingerprint pattern. It removes low frequency components along the direction orthogonal to the ridge direction. One example of the preprocessed image is shown in Figure 4:

(1) $$I_{H}(x,y)=I(x,y)-\frac{1}{k^{2}}\sum_{i=1}^{k}\sum_{j=1}^{k}I(i,j)+b$$

(2) $$I_{L}(x,y)=\frac{1}{2}(\frac{1}{m^{2}}\sum_{i=1}^{m}\sum_{j=1}^{m}I_{H}(i,j)+\frac{1}{n^{2}}\sum_{i=1}^{n}\sum_{j=1}^{n}I_{H}(i,j))$$

### 2.2. Descriptors Extraction

Based on the image processing of the previous sub-section, binarization and thinning are performed. Minutiae are detected from the thinning image. The type of minutiae can also be classified into ridge bifurcation and ridge ending. A ridge ending minutia is a point where a ridge terminate, while a ridge bifurcation minutia is a point where a ridge splits from a single path to two paths. The minutia *m* is defined by Equation (3), which includes its *x* coordinate, *y* coordinate and the direction by tracing.

(3) $$D_{M}(m)=(x_{m},y_{m},\theta_{m})$$

**Figure 5.**
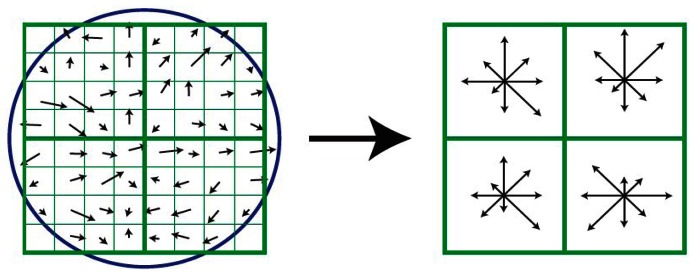
SIFT Descriptor.

The SIFT descriptor are calculated based on the processed image in Section 2.1. The skeleton image should not be used to extract minutiae because the texture information needed by the SIFT operator are removed in the skeleton image. A SIFT descriptor [10,11] is proposed by computing the gradient magnitude and orientation at each point in a region around the sampling point as shown on the left of Figure 5. These samples are then accumulated into orientation histograms summarizing the contents over the sub-regions as shown on the right of Figure 5. The proposed descriptor consists of the SIFT descriptor of the minutia and SIFT descriptors at several sampling points around the minutia.

**Figure 6.**
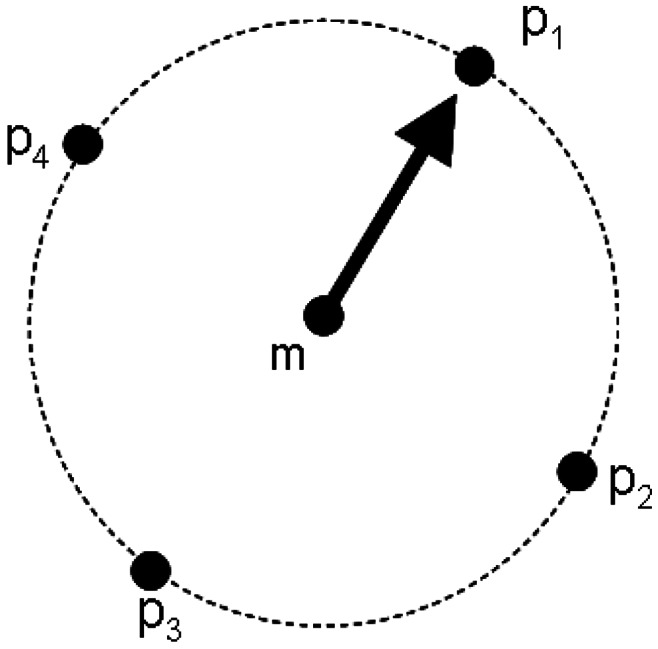
SIFT-based Minutia Descriptor (SMD).

Let *m* and *p_i_* denote the detected minutia and the sampling point. The descriptor of detected minutia is defined as:

(4) $$\text{D}(m)=\{S(m),{\left\{S(p_{i})\right\}}_{i=1}^{d}\}$$

where *S(m)* denotes the SIFT descriptor of minutia, *d* denotes the number of sampling points and *S(p_i_)* denotes the SIFT descriptor of sampling point around minutia *m*. Figure 6 shows the structure when *d* equals 4.

In our previous work [18], it has been shown that the SIFT descriptor around minutiae points plays a major role when SIFT algorithm is applied to fingerprint verification. Therefore, with this definition, we believe that the texture information of the region around minutia can be included in this descriptor. The selection of parameter *d* is a tradeoff between the verification accuracy and speed. The original SIFT extraction can extract over 2,000 key points from a fingerprint image [15]. It will require heavy computational resources for both extraction and verification. According to our experiments, *d* is selected as 4. The number of the SIFT descriptors of the proposed extraction is 5 × *M* if *M* denotes the number of minutiae of the fingerprint image. In other words, the number of the SIFT descriptors of one fingerprint image is only about 50∼200, which can decrease the computation complexity significantly.

## 3. Improved All Descriptor-Pair Matching (iADM)

Using the proposed SIFT-based minutia descriptor (SMD), we developed a two-step fast matching method, called improved All Descriptor-Pair Matching (iADM). Compared with previous work [15], it can significantly improve the accuracy and simplify the computation complexity.

### 3.1. Optimized ADM

In the previous work [15], the algorithm was based on point-wise matching, followed by the trimming of false matches. Point-wise matching in [15] rejects the closest neighbor if the ratio of the *Euclidean Distance* of the closest neighbor (*d_1_*) and the second closest neighbor *(d_2_)* is too high (higher than the threshold). For the trimming of false matches, two fingerprint images were placed side by side, and lines between each matched pair were connected. The orientation and length of the connected lines in majority are collected as a standard. The matches having orientations and lengths different from the standard are trimmed. The matching score is calculated by counting the lines which are similar with the standard.

Two optimizations are performed based on ADM. Firstly the point-wise matching rejects a large number of genuine descriptor pairs because of the similar parallel ridge shapes. Secondly the trimming of false matches assumes that the fingerprint images are limited to very small rotations, so it would not work properly if the fingerprint images have large rotations. Therefore, the ADM can be optimized by employing the Hough Transform for all closest pairs calculated by *Euclidean Distance.* The Hough Transform is performed on the 2D location and orientation. In practice, there is only one shift vector (*Δx*, *Δy*) between the source descriptor (*s_x_*, *s_y_*) and the closest neighbor (*c_x_*, *c_y_*) if we rotate source image by *Δ_θ_*, as shown in Equation (5). Therefore, to evaluate accumulation of similarity measure at a rotation, 2-D accumulator array ***B*** is sufficient. The best alignment at a fixed rotation is found by searching for the maximum similarity score in array ***B***. Similarly, the best alignment (b_Δx_, b_Δy_, b_Δθ_) between two images can be calculated by simply selecting the alignment which has the maximum similarity among the full rotations.

(5) $$\left[\begin{array}{l} \Delta x\\ \Delta y \end{array}\right]=\left[\begin{array}{l} c_{x}\\ c_{y} \end{array}\right]-\left[\begin{array}{ll} \cos\Delta \theta & -\sin\Delta \theta \\ \sin\Delta \theta & \cos\Delta \theta \end{array}\right]\left[\begin{array}{l} s_{x}\\ s_{y} \end{array}\right]$$

Suppose the size of the image is 256 × 256, the quantization factor for 2-D Hough Transform is 4, the memory requirement for accumulator ***B*** would be ((256 × 2) × (256 × 2) / 4) / 4 = 16, 384 memory units. In our experiment, the orientation is quantized into 20 different directions. Compared with ADM, the optimized ADM can improve the performance of accuracy significantly but with the sacrifice of only about 16 K words memory. The accuracy performance improvement is shown in the experiment section.

### 3.2. Improved ADM

The optimized ADM works well for an accurate matching, but it may still suffer from the limitation of computation time when one-to-many verifications is required. Therefore, based on the optimized ADM, we propose the improved All Descriptor-Pair Matching (iADM), which is two-step fast matching method consisting of global search and local search.

**Figure 7.**
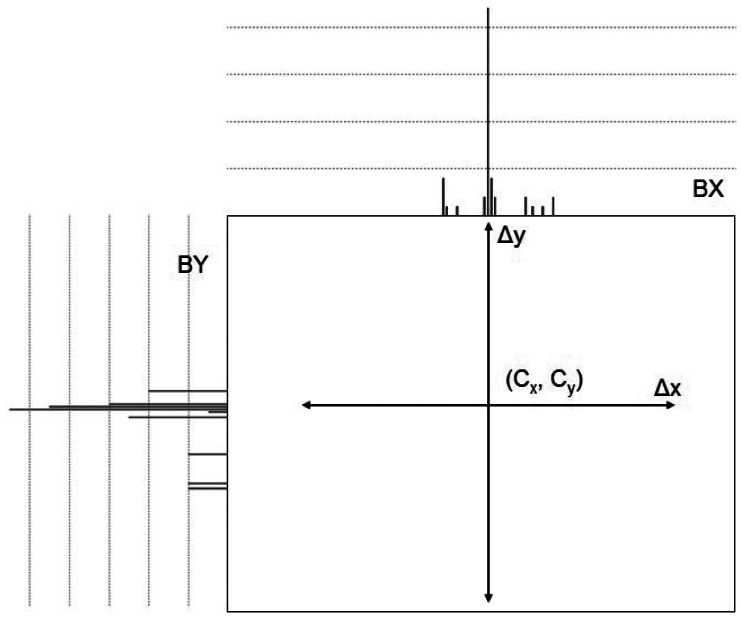
Distributions of two 1-D arrays in the first step.

In the first step, the optimized ADM is performed only based on the SIFT descriptor of minutiae positions. In other words, only the *S(m)* of *D(m)* is used in Equation (4). As the result of this step, the peak of the Hough Transform which shows the best alignment transformation (*Δx, Δy, Δθ*) is obtained. This is the result of the rough alignment transformation which will be used for local search. Because the SIFT descriptor of minutiae positions is only *N*/*5* where *N* is the number of SIFT points, the calculation of *Euclidean Distance* is (N^2^/25) times. In practice, a more efficient way can be applied. Instead of using 2-D accumulator array, two 1-D accumulator arrays, BX and BY, are used as the histogram arrays for each corresponding axis of the displacement vector. As shown in Figure 7, these histograms accumulate scores for each axis, *Δx* and *Δy.* Then the center of the distribution score, *C_x_* and *C_y_*, can be obtained by analyzing these histograms. The simplest way is to extract the locations with the maximum value of each histogram. The best rotation *Δθ* can also be obtained by repeating all possible rotation steps by Optimized ADM. The method of employing two 1-D arrays has two advantages. It can decrease the time for searching peak from the accumulator array. Moreover, the memory requirement can also be decreased.

**Figure 8.**
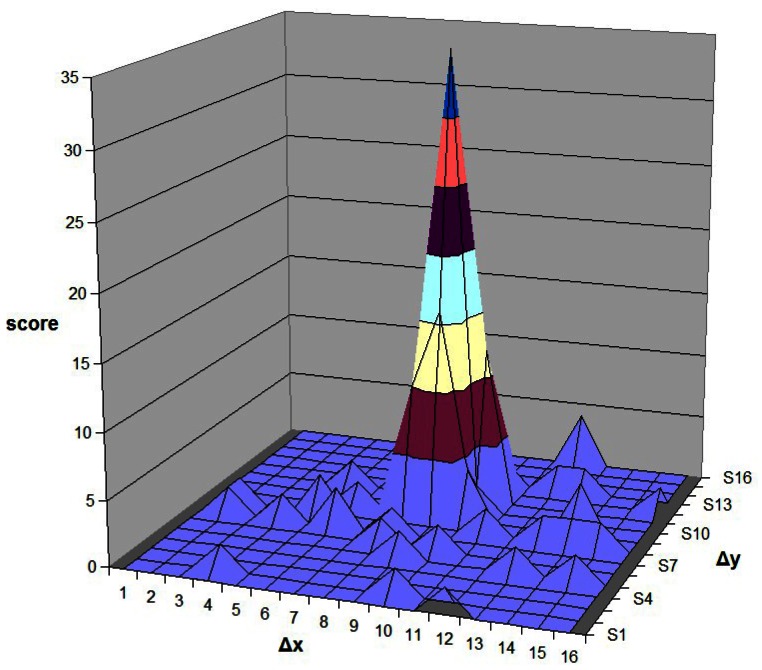
Distributions of small 2-D accumulator array in the second step.

In the second step, the local search is applied. Only the SIFT descriptor of sampling points around minutiae are used for local matching. In the similar way, the matching is performed by employing the Hough Transform for all closest pairs calculated by *Euclidean Distance* in this local area. A small 2-D accumulator array *C*, with (*C_x_, C_y_*) as the center, is employed to accumulate the score. The descriptor pairs that have displacement vectors outside of *C* are not required to calculate the *Euclidean Distance*. Notably, there are four sampling SIFT descriptors for each minutia which are rotation invariant. Therefore, the sampling SIFT descriptor only match with the descriptors of its own class. For example, {*S_0_(m_0_), S_0_(p_1_), S_0_(p_2_), S_0_(p_3_)*} belong to one minutia of Image #0 while {*S_1_(m_0_), S_1_(p_1_), S_1_(p_2_), S_1_(p_3_)*} belongs to one minutia of Image #1. S_0_(p_1_) only needs to match with S_1_(p_1_), the *Euclidean Distance* computation of both (*S_0_(p_1_), S_1_(p_2_)*) and (*S_0_(p_1_), S_1_(p_3_)*) are not required. The distribution of scores in the array *C* is obtained as shown in Figure 8. The value of the peak is searched from this distribution of scores and considered as the final matching score. In our experiment, the size of *C* is selected as 16.

### 3.3. Computational Complexity

The iADM can achieve almost the same accuracy as the optimized ADM while decreasing the computation complexity significantly. Suppose the average number of descriptors of fingerprint image is 5 × *M* if *M* denotes the number of minutiae of the fingerprint image, the calculation times of *Euclidean Distance* using ADM would be 25 × *M^2^*. In iADM, the calculation of *Euclidean Distance* in the first step is *M^2^* for rough alignment, while in the second step the calculation time is *n* times 4 × *M* where *n* is related to the size of the small 2-D accumulator array *C.* According to the experiments, the average value of *n* is 3. Suppose the average number of minutiae in one fingerprint image is 30, the iADM could be around 18 times faster than ADM.

## 4. Experimental Results

### 4.1. Database and Parameters

In order to demonstrate the performance of FISiA, several well-known fingerprint databases were tested. Database DB_swipe was captured using a swipe sensor with a width of only 0.25 inches (only 126 pixels in the horizontal direction for 500 DPI). Another database is DB_cuts, which includes fingerprint images with plenty of broken ridges attributable to cuts, which were captured by a capacitive area sensor. Moreover, to compare FISiA with other SIFT-based algorithms in the [15] and those of previous studies utilizing minutiae and additional data [6,7,9,17], experiments on the standard FVC2002 database were conducted as well. The descriptions of the databases are given in Table 1.

False Match Rate (FMR), False Non-Matching Rate (FNMR) and Equal Error Rate (EER) are important factors to estimate the performance of a fingerprint identification system. The FMR is the rate at which the system incorrectly accepts imposter fingerprint inputs. FNMR is the rate at which inputs of genuine fingerprint are incorrectly rejected by the system. EER is the rate at which both FMR and FNMR are equal. In the experiments, EER, FMR10000 and ZeroFMR are used to estimate the accuracy of the system. FMR10000 and ZeroFMR are defined as the values of FNMR when FMR is 0.01% and zero respectively.

### 4.2. Evaluation for Narrow-Swiped Fingerprints

**Figure 9.**
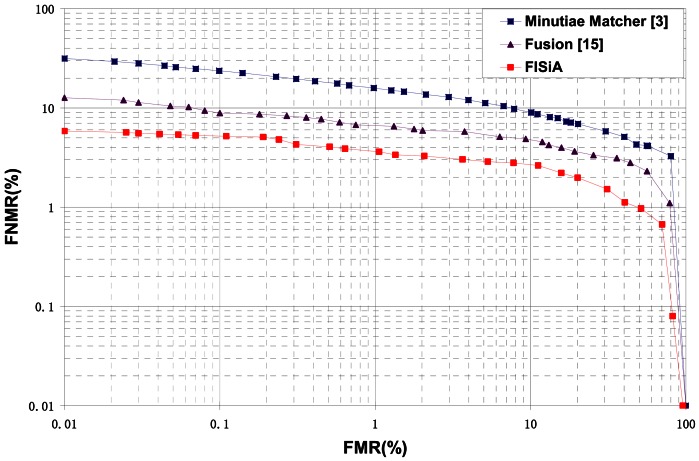
Comparison on a specific database, DB_Swipe.

**Figure 10.**
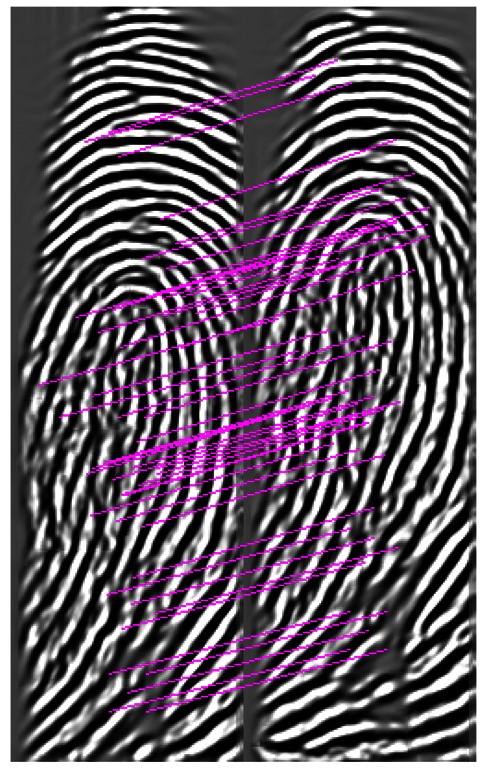
Example of genuine pairs in DB_Swipe.

The first private database comprises images captured by a narrow-width swiped sensor. The width of this sensor is only 126 pixels. Therefore, the number of minutiae for each fingerprint image in this database is only 12 on average. Moreover, this database was collected intentionally with large shift when successively sliding the fingers. Therefore, the FMR10000 of the minutiae matcher increased to 31.5%. The fusion of SIFT method and minutia-based method proposed in [15] is implemented with the same preprocessing for comparison. It is observed from Table 2 that FISiA can achieve a much better result compared with both the minutiae matcher and the fusion in [15]. The rich information on the ridge pattern collected by the SIFT contributes substantially for this significant improvement. The Receiver Operating Characteristic (ROC) curves obtained by the minutiae-based matcher and FISiA are shown in Figure 9. A pair of fingerprints for matching is shown in Figure 10 as examples to demonstrate the advantages of our proposed FISiA.

### 4.3. Evaluation for Fingerprints with Cuts

**Figure 11.**
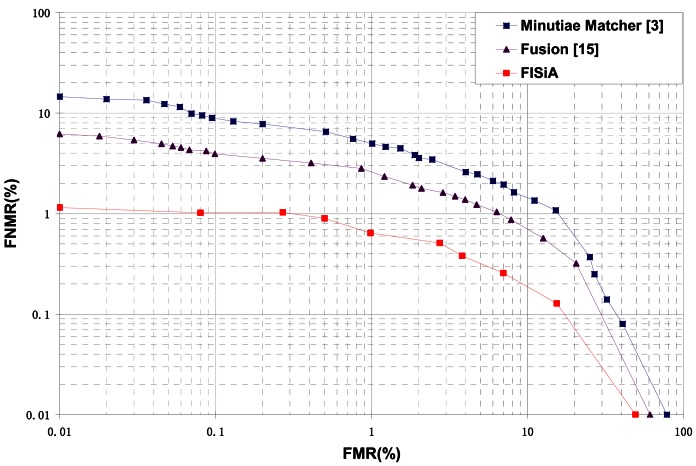
Comparison on a specific database, DB_cuts.

**Figure 12.**
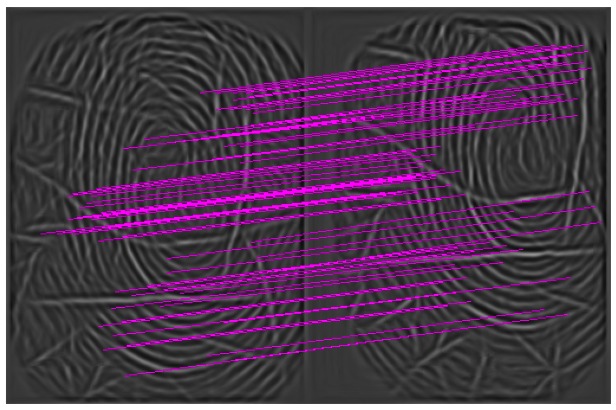
Example of genuine pairs in DB_cuts.

The second private database comprises fingerprints with lots of cuts. Table 3 shows the results of FMR10000 on this database tested using the minutiae matcher, fusion in [15] and FISiA. The ROC curve is shown in Figure 11. The performance of FISiA shows the robustness to the noises in this cuts database. The cuts, which shorten minutiae ridge shapes, result in low accuracy for the minutiae matcher. Moreover, the cuts allow the SIFT descriptor to be more distinctive because this descriptor uses the gradient information of a region around the key point location. In other words, the cuts and broken ridges become an important part of the features. One example is shown in Figure 12, in which the matched key points are marked by red lines, including the broken ridges.

### 4.4. Comprehensive Comparison

**Figure 13.**
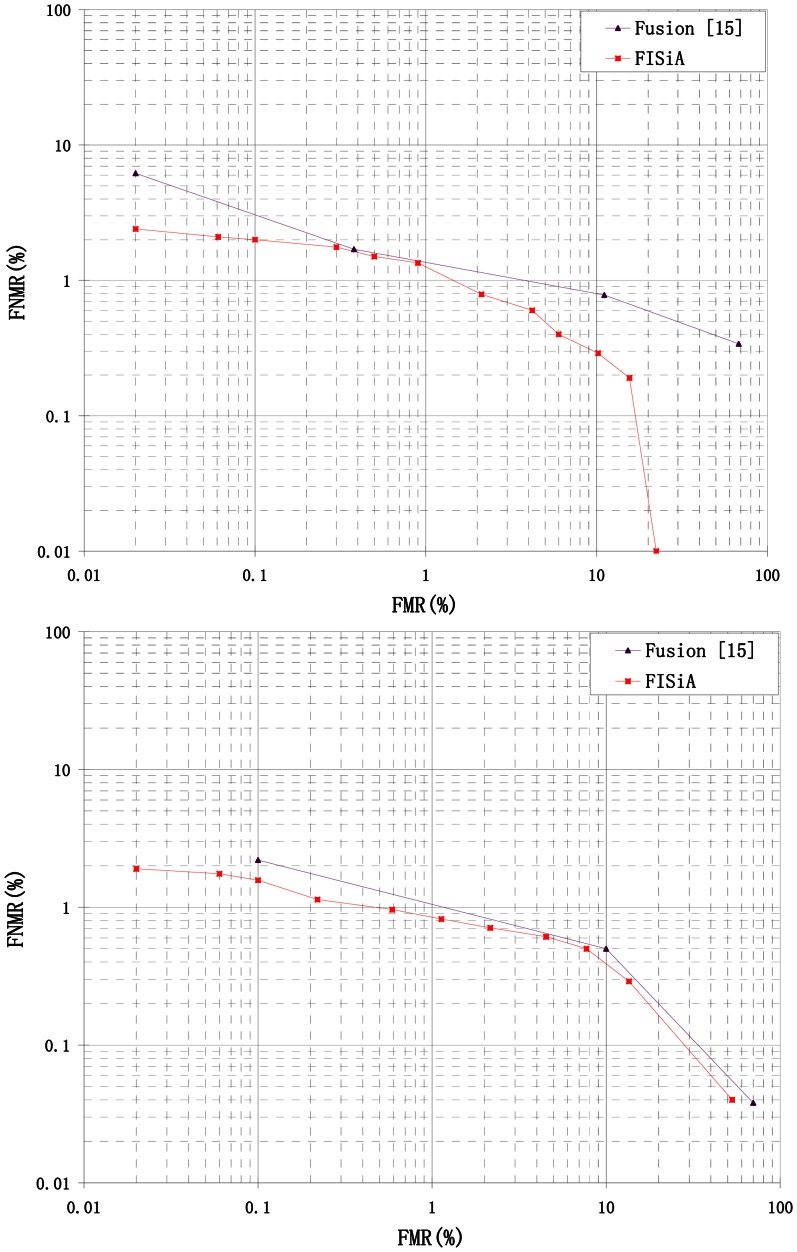
ROC curves of Fusion [15] and FISiA on FVC2002 DB1 and DB2.

FISiA is designed for the purpose of specific fingerprint authentication. In order to thoroughly compare with the fusion method in [15], another experiment was performed on the FVC2002 DB1 and DB2 according to the standard guideline given in [19]. The total matching on the database comprised of 2,800 genuine matches and 4,950 imposter matches. The comparison of error matching rates among FISiA and the fusion method of SIFT and minutia-based method in [15] are presented in Table 4. The average matching speed, including feature extraction is also given. The ROC curves obtained on FVC2002 DB1 and DB2 using Reference [15] and FISiA are shown in Figure 13. The improvement in the error rate of FISiA is attributable to proper image processing, descriptor definition and thew optimization of the original SIFT matcher, *i.e.*, SIFT-based Minutia Descriptor (SMD). The improvement in the speed of FISiA can be resulted from the decrease of the number of descriptors and the strategy of two-step fast matching, *i.e.*, improved All Descriptor-Pair Matching (iADM).

**Figure 14.**
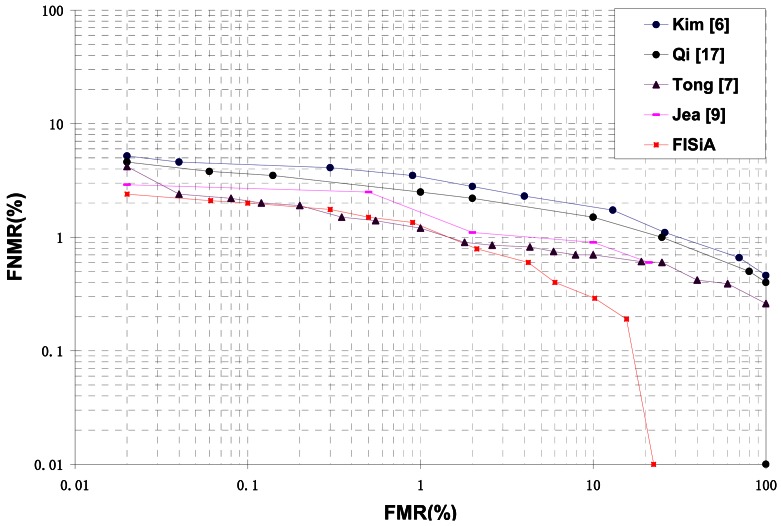
ROC curves of FISiA and other related methods on FVC2002 DB1.

A number of previous studies [6,7,9,17] that utilize both minutiae and additional data to improve accuracy are discussed in the introduction. The ROC curves of FISiA and other related methods on FVC2002 DB1 are shown in Figure 14. Experimental results showed that our proposed FISiA is not only suitable for the specific fingerprints, but also can deal with the normal fingerprints.

The execution time for FISiA for feature extraction is about 0.1 s∼0.15 s and the time required for a single verification is about 1 ms∼15 ms with a 2.0 GHz PC. This duration is slower than that of the conventional minutiae-based algorithm [3]. However, a significant improvement on accuracy for specific fingerprint images has been achieved by FISiA, which can be suitable for identification on smart phones and PCs.

In practice, if the narrow-swiped sensor is used, the proposed algorithm can be employed directly. If the sensor with normal size is used, since the fingerprint with lots of cuts is only a small percentage of the database, it is recommended that an estimation of fingerprint quality such as [20] can be performed in the beginning. Only the fingerprints with lots of cuts need to apply FISiA for better performance as the conventional minutiae matcher is accurate enough for the fingerprints without lots of cuts.

## 5. Conclusions

In this paper, a fingerprint identification algorithm (FISiA) using SIFT-based Minutia Descriptor (SMD) and improved All Descriptor-Pair Matching (iADM) is presented. FISiA can deal not only with normal fingerprints, but also specific fingerprints with lots of cuts and less overlapping areas. FISiA employed image processing to make the descriptor more robust to variations in finger pressure or differences in skin characteristics. SMD is proposed to improve the SIFT-based algorithm through image processing, descriptor extraction and matcher. Furthermore, a two-step fast matcher, *i.e.*, iADM, is proposed to decrease the computational complexity. Experimental results on well-known databases indicate a significant improvement compared with the conventional minutiae matcher and other recent methods. FISiA could be applied as a universal or specific fingerprint authentication system dealing with different fingerprints obtained by a variety of sensors.

## Figures and Tables

**Table 1.** Description of Databases.

**Database**	**Sensor Type**	**Image Size**	**Image Number**	**Resolution (DPI)**
DB_swipe	Optical	126 × 400	200 × 10	500
DB_cuts	Capacitive	256 × 336	100 × 13	500
FVC2002	Optical	388 × 374	100 × 8	500

**Table 2.** Comparison between the minutiae matcher and FISiA on DB_Swipe.

**Matcher**	**FMR10000**
Minutiae Matcher [3]	31.5%
Fusion [15]	12.7%
FISiA	5.9%

**Table 3.** Comparison between the minutiae matcher and FISiA on DB_cuts.

**Matcher**	**FMR10000**
Minutiae Matcher [3]	14.6%
Fusion [15]	6.2%
FISiA	1.2%

**Table 4.** Comparison with other SIFT-based fingerprint matcher on FVC2002.

**Database**	**SIFT-Based Algorithm**	**EER**	**ZeroFMR**	**Matching Time**
DB1	Fusion [15]	0.99%	6.2%	1.8s
	FISiA	1.0%	2.4%	0.01s

DB2	Fusion [15]	1.07%	2.2%	2.5s
	FISiA	0.89%	1.9%	0.015s
